# ALV-J inhibits autophagy through the GADD45β/MEKK4/P38MAPK signaling pathway and mediates apoptosis following autophagy

**DOI:** 10.1038/s41419-020-02841-y

**Published:** 2020-08-12

**Authors:** Zhihong Liao, Xinheng Zhang, Cailiang Song, Wencheng Lin, Yuzhen Cheng, Zi Xie, Sheng Chen, Yu Nie, Aijun Li, Huanmin Zhang, Hongxin Li, Haiyun Li, Qingmei Xie

**Affiliations:** 1 Lingnan Guangdong Laboratory of Modern Agriculture & Guangdong Provincial Key Lab of Agro-Animal Genomics and Molecular Breeding, College of Animal Science, South China Agricultural University, 510642 Guangzhou, PR China; 2 Key Laboratory of Animal Health Aquaculture and Environmental Control, Guangdong, 510642 Guangzhou PR China; 3 South China Collaborative Innovation Center for Poultry Disease Control and Product Safety, 510642 Guangzhou, PR China; 4 Guangdong Engineering Research Center for Vector Vaccine of Animal Virus, 510642 Guangzhou, PR China; 5 College of Science and Engineering, Jinan University, 510632 Guangzhou, PR China; 6 USDA, Agriculture Research Service, Avian Disease and Oncology Laboratory, East Lansing, MI 48823 USA

**Keywords:** Macroautophagy, Apoptosis, Infection

## Abstract

Autophagy and apoptosis, which are important processes for host immunity, are commonly exploited by viruses to facilitate their survival. However, to the best of our knowledge, very few studies have researched the mechanisms of action of the autophagic and apoptotic signaling pathways following viral infection. Thus, the present study aimed to investigate the mechanisms of action of growth arrest and DNA-damage-inducible β (GADD45β), an important resistance gene involved in the host resistance to ALV-J. Both ALV-J infection and the overexpression of GADD45β inhibited autophagy during the early stages, which prevented the autophagosomes from binding to the lysosomes and resulted in an incomplete autophagic flux. Notably, GADD45β was discovered to interact with MEKK4 in DF-1 cells. The genetic knockdown of GADD45β and MEKK4 using small interfering RNA-affected ALV-J infection, which suggested that ALV-J may promote the binding of GADD45β to MEKK4 to activate the p38MAPK signaling pathway, which subsequently inhibits autophagy. Furthermore, ALV-J was revealed to affect the autophagic pathway prior to affecting the apoptotic pathway. In conclusion, to the best of our knowledge, the present study was the first to investigate the combined effects of ALV-J infection on autophagy and apoptosis, and to suggest that ALV-J inhibits autophagy via the GADD45β/MEKK4/p38MAPK signaling pathway.

## Introduction

Autophagy is an evolutionarily conserved catabolic process involving the self-degradation of organelles and cytosolic macromolecules. It has been discovered to share a complex and multifaceted functional relationship with apoptosis. Autophagy can either inhibit or enhance apoptotic cell death depending on the cell type, environment or the manner of stimulation. The crosstalk between autophagy and apoptosis is complicated; autophagy and apoptosis can coexist or occur sequentially in numerous different circumstances^1^, which is thought to be triggered by common upstream signals that result in the combined activation of autophagy and apoptosis. For example, autophagy can be initiated through the interaction between various molecules, including Bcl-2, Beclin-1, Atg5, and Atg12, which subsequently results in the activation of the intrinsic apoptosis pathway^2–4^. However, autophagy and apoptosis may also remain mutually exclusive under certain conditions; for instance, Atg12 can bind with Atg3 to regulate mitochondrial homeostasis and apoptosis, without involving autophagy^5^. In other circumstances, apoptosis may be induced following autophagy, or autophagy can often culminate with the inhibition or a blockade of caspase activity^6^. However, autophagic processes are not directly responsible for activating apoptosis, as the effect is most likely to be indirect.

Viruses have evolved various mechanisms to avoid or exploit autophagic processes to promote their survival or aid their infection at different stages of the viral life cycle^7^. Increasing evidence has suggested that autophagy may serve as a platform for viral replication, such as for the classical porcine reproductive and respiratory syndrome virus, swine fever virus, and rotavirus^8,9^. In fact, autophagy has been reported to have both anti-viral and pro-viral roles in the life cycle and pathogenesis of different types of virus^10,11^, thus autophagy is both important for viral surveillance, but it may also serve as an effector^12^. Since establishing that autophagy serves a complex role in HIV infection, accumulating evidence has indicated that HIV requires the early stages of autophagy for the virus to undergo its early replication steps and the later stages of autophagy to protect itself from degradation^13^. In fact, numerous HIV proteins have been implicated in the regulation of autophagy, by either inhibiting or stimulating autophagy through directly interacting with autophagy-related proteins^14^. For example, the accessory protein NEF was found to activate autophagy by sustaining the assembly of the ULK1/Beclin-1/ATG16 complex during HIV infection^15^.

J subgroup avian leukemia is a tumor-causing infectious disease caused by the J subgroup avian leukemia virus, which mainly infects bone marrow cells, and metastasizes to liver, kidney, spleen and so on. In fact, it can cause tumors, immunosuppression and decline in production performance, which result significant economic losses in the poultry industry. To date, no vaccines or drugs have been developed to treat the disease effectively. On the other hand, ALV-J, like human immunodeficiency virus and bovine foamy virus, belongs to the retrovirus family of the retroviridae family and it shares a similar replication cycle to HIV-1. A previous study found that ALV-J inhibited autophagy to promote the replication of the virus itself^16^. In addition, ALV-J infection was discovered to affect the mTOR signaling pathway and activate the phosphorylation of AKT^17–20^. However, its specific mechanisms of action remain unclear. Therefore, the present study aimed to investigate the methods by which ALV-J can negatively regulate autophagy. Simultaneously, it provides new research ideas for the replication of other retroviruses.

Growth arrest and DNA-damage-inducible beta (GADD45β) belongs to a family comprising of GADD45α, GADD45β/Myd118, and GADD45γ, whose transcript levels increase following stressful growth arrest conditions^21^. The proteins encoded by this gene family have been demonstrated to serve an important role in inhibiting cell growth and apoptosis^22,23^. In a previous study, GADD45β mediated the activation of mitogen activated protein kinase (MAPK) in Th1 cells to affect the production of γ-interferon, which served a crucial role in mediating the tumor suppressor effect^24^. It is well established that GADD45β has a close relationship with the MAPK signaling pathway, including MAPK1-MAPK3 (ERK2-ERK1), JNK, and p38MAPKs, which are all involved in the regulation of autophagy and apoptosis. In fact, MAPK/ERK kinase kinase 4 (MEKK4), apoptosis signaling kinase 1 (ASK1), and MAP kinase kinase (MKK)7 have all been discovered to be GADD45β interaction partners^25^. Previous studies have demonstrated that autophagy was inhibited following the activation of the GADD45β/MEKK4/p38MAPK signaling pathway^26^. Furthermore, our previous study revealed that GADD45β was highly expressed in ALV-J resistant chickens, which also affected the replication of ALV-J in DF-1 cells^27^. Based on these previous findings, the current study further investigated and determined whether ALV-J may regulate autophagy through GADD45β.

p38MAPK, first described in 1994, is activated by environmental and genotoxic stresses;^28^ the upstream action of MKK3, MKK6 kinases, and MKK4 can promote the phosphorylation of p38MAPK by interacting with related proteins^29^. p38MAPK has been found to be a major determinant of the balance between p53-dependent apoptosis and autophagy^30,31^. In addition, sufficient evidence has proved that p38MAPK may inhibit starvation-induced autophagy through the phosphorylation of Atg5^26^. The interaction between mAtg9 and p38IP negatively regulated both basal autophagy and starvation-induced autophagy through p38MAPK^32^. Thus, these findings suggested that p38MAPK may be important for the transition from autophagy to apoptosis. Although p38MAPK has a dual role in the regulation of autophagy and apoptosis, how p38MAPK may control the balance between apoptosis and autophagy remains poorly understood, alongside how p38MAPK-regulated autophagy promotes the viral survival mechanism.

To the best of our knowledge, the present study observed for the first time that ALV-J could inhibit autophagy and induce incomplete autophagy in DF-1 cells. Furthermore, increased expression levels of GADD45β were discovered in cells following the infection with ALV-J, and ALV-J infection was observed to activate the p38MAPK/JNK signaling pathway. These findings highlighted the potential effect of the GADD45β/MEKK4/p38MAPK signaling pathway on ALV-J-mediated autophagy. Thus, the present study suggested that autophagy may precede apoptosis during ALV-J infection.

## Materials and methods

### Antibodies and reagents

Rabbit anti-LC3B (cat. no. L7543), anti-SQSTM1 (cat. no. P0067), anti-GADD45β (cat. no. AV48346), and anti-LAMP-1 (cat. no. SAB3500285) primary antibodies were purchased from Sigma-Aldrich; Merck KGaA, whereas the anti-MEKK4 primary antibody (cat. no. sc166197) was purchased from Santa Cruz Biotechnology, Inc. Rabbit anti-Atg5 (cat. no. 12994S), anti-JNK1 (cat. no. 3708S), anti-p-JNK (cat. no. 4668T), anti-p38MAPK (cat. no. 8690S), anti-phospho-p38MAPK (cat. no. 4511S), anti-Flag (cat. no. 14793S), anti-β-actin (cat. no. 4970S), and anti-HA (cat. no. 2367S) were purchased from Cell Signaling Technology, Inc.; LysoTracker^™^ Red DND-99 (cat. no. L7528), ProLong Gold Antifade (cat. no. P36941), Lipofectamine^®^ 3000 reagent (cat. no. L3000015), goat anti-rabbit IgG (H+L) secondary antibody and DyLight 405 (cat. no. 35551) were purchased from Invitrogen; Thermo Fisher Scientific, Inc. Ad-mCherry-GFP-LC3B (cat. no. C3011) was purchased from Beyotime Institute of Biotechnology, and rapamycin (Rapa; cat. no. S1039) and SP600125 (cat. no. S1640) were purchased from Selleck Chemicals. Finally, SB203580 (cat. no. S8307) was purchased from Sigma-Aldrich; Merck KGaA, the horseradish peroxidase-conjugated goat anti-mouse IgG (H+L) secondary antibody (cat. no. SA00001) was purchased from ProteinTech Group, Inc. and the rabbit polyclonal antibodies to the gp37 proteins of ALV-J were purchased from Sangon Biotech Co., Ltd.

### Cell culture and viruses

DF-1 cells were purchased from the American Type Culture Collection. Cells were cultured in DMEM, supplemented with 10% FBS, and maintained at 37 °C and 5% CO_2_.

For the preparation of chicken embryo fibroblasts (CEF) cells cultures: 9–11-day-old chicken embryo was chosen and sterile techniques were used to open shell and take out the embryo, followed with remove heads, limbs and internal organs, then added an appropriate amount of 0.25% trypsin solution incubate slowly in a 37 °C incubator for 15 min, finally used 0.22-μm cell sieve and count. The prepared cells were cultured in DMEM, supplemented with 10% FBS, and maintained at 37 °C and 5% CO_2._

The ALV-J strain, SD1005, was provided by Professor Cui from Shandong Agricultural University. Briefly, DF-1 cells and CEF cells were infected with ALV-J in DMEM. Following 4 h of incubation, the media was replaced with DMEM, supplemented with 2% FBS. For certain experiments, cells were stimulated with a suitable concentration of inducer or inhibitor for 2 h prior to ALV-J virus infection.

### Construction of expression plasmids and cell transfection

GFP-LC3 was stored in our laboratory. To construct the pRK5-flag-GADD45β and pRK5-HA-MEKK4 plasmids, GADD45β and MEKK4 mRNA were amplified using PCR. Briefly, total RNA was extracted from DF-1 cells. PCR was subsequently performed with the following primer pairs: GADD45β forward, 5′-GGATCCATGACTCTGGAAGAGACGCA-3′ and reverse, 5′-GTCGACTCACTCAGGTAAGGCAATAGTTGG-3′; and MEKK4 forward, 5′-AGCTTTGCCAGAAAGTGGATGAACTACGTGCTAACAAAATGTGAGAGAGTGGCCGA-3′ and reverse, 5′-CAAGTAAAACCTCTACAAATGTGGTA TGGCTGATTATGATCAGTTATCTAGATCCGGTTCATTCTTCA-3′. The cDNA of GADD45β and MEKK4 were cloned into the pRK5-flag and pRK5-HA vectors, respectively, and the predicted sequences were successfully identified by digestion. Cells were transfected with these plasmids using Lipofectamine^®^ 3000 reagent, according to the manufacturer’s protocol.

Small interfering RNA (siRNA) targeting GADD45β (5′-CCAGAUAACGUGGCGUUCUTT-3′), MEKK4 (5′-GCGAAUAAUGGAACUGCUATT-3′) and the negative control (5′-UUCUCCGAACGUGUCACGUTT-3′) were synthesized by Shanghai GenePharma Co., Ltd. The siRNAs were transfected into DF-1 cells at a concentration of 2 μg/well in a six-well plate. The efficiency of siRNA knockdown was analyzed using western blotting and specific antibodies.

### Reverse transcription-quantitative PCR (RT-qPCR)

Total RNA was extracted from DF-1 cells by using Trizol reagent. Total RNA was reverse transcribed to cDNA using a PrimeScript RT Reagent kit (cat. no. AK2601; Takara Bio, Inc.). qPCR was subsequently performed using an ABI 7500 Sequence Detection system. Expression levels were quantified using the 2^−ΔΔCq^ method and normalized to GAPDH.

### Western blotting

Total protein was extracted from DF-1 cells following treatment. Total protein was quantified using a bicinchoninic acid assay kit (cat. no. CW0014). The membranes were incubated with the aforementioned primary antibodies (1:1,000). Protein bands were visualized using a C600 ultra-sensitive chemiluminescence imager and expression levels were quantified using Image-Pro Plus software. Each experiment was performed independently in triplicate.

### Caspase-3 and caspase-8 activity assays

Caspase-3 and Caspase-8 activities were analyzed using their corresponding kits (cat. nos. C1116 and C1151, respectively; Beyotime Institute of Biotechnology). Briefly, total protein was extracted from DF-1 cells and quantified using a Bradford Protein Concentration assay kit (cat. no. P0006; Beyotime Institute of Biotechnology). Protein extracts were incubated with reagent from Caspase-3 and Caspase-8 activities kit at 37 °C for 2 h in the dark and detected at a wavelength of 405 nm using a MultiScan Go microplate reader (Thermo Fisher Scientific, Inc.).

### Confocal microscopy

Cells were transfected with 1 μg DNA plasmid using Lipofectamine^®^ 3000 reagent for 24 h in a laser confocal culture dish (cat. no. 80100215). To stain the acidic compartments, live cells were stained with 50 nM LysoTracker Red and incubated for 2 h at 37 °C in the dark. Following the incubation, DMEM was removed and cells were subsequently washed with TBST, fixed with 4% paraformaldehyde, permeabilized with 0.1% Triton X-100 and blocked with 5% BSA (cat. no. AAPR305) at 4 °C overnight. Cells were incubated with the monoclonal or polyclonal antibodies to the target proteins and the corresponding Alexa-Fluor-conjugated secondary antibodies. Then, ProLong Gold Antifade was plated in the laser confocal culture dish to stain the nucleus and prevent the fluorescence quenching. Stained cells were subsequently analyzed using a Leica SP8 STED3X confocal laser scanning microscope (Leica Microsystems GmbH).

### Transmission electron microscopy (TEM)

DF-1 cells were plated into six-well cell culture plate and following 24 h of incubation, cells were harvested and fixed in 2.5% glutaraldehyde (cat. no. AAPR46) for 24 h at 4 °C. Subsequently, cells were rinsed with buffer, fixed with citric acid and dehydrated in a series of ethanol. Then, cells were permeabilized with different ratios of ethanol: resin permeation and embedded with pure resin, which was polymerized at 70 °C. Stained cells were visualized using a transmission electron microscope (Hitachi, Ltd) to observe the various structures within the cells.

### Co-immunoprecipitation (Co-IP)

Co-IP was performed as previously described. Briefly, the transfected DF-1 cells were washed twice with cold PBS and total protein was extracted using RIPA lysis buffer (cat. no. CW2333; CoWin Biosciences), supplemented with protease inhibitor cocktail (cat. no. CW2000; CoWin Biosciences) for 10 min. The soluble fractions were incubated with anti-Flag immunomagnetic beads (cat. no. B26102; Biomake.cn) for 2 h at 4 °C. Subsequently, the pretreated supernatants were further incubated with an anti-Flag antibody on an orbital shaker overnight at 4 °C. The bound proteins were eluted following boiling in 2× SDS-PAGE loading buffer and detected using western blotting.

### Statistical analysis

Statistical analysis was performed using GraphPad Prism 5 software (GraphPad Software, Inc.). Statistical differences between different groups were determined using a two-way ANOVA. *P* < 0.05 was considered to indicate a statistically significant difference.

## Results

### ALV-J infection increases the expression levels of GADD45β

**Fig. 1:**
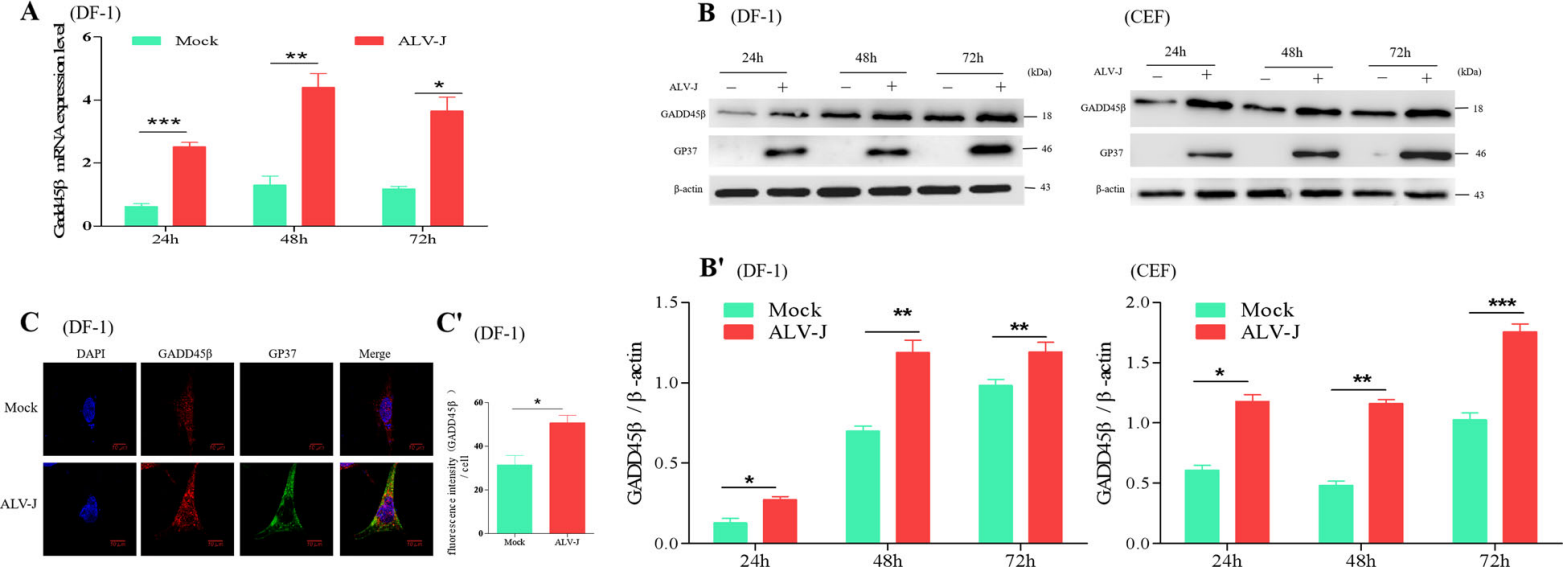
GADD45β expression levels increase in DF-1 cells following ALV-J infection. **a** DF-1 cells were infected with ALV-J for 24, 48, or 72 h and the expression levels of GADD45β were analyzed. Statistical differences were determined using two-way ANOVA. ^*^P < 0.05; ^**^P < 0.01; ^***^P < 0.001. **b** Following 24, 48, or 72 h of ALV-J infection, total protein was extracted from DF-1 cells or CEF cells, the expression levels of GADD45β were analyzed using western blotting. The chart (**b′**) shows the quantification of GADD45β/β-actin in (**b**). **c** Cells were infected with ALV-J for 24 h and immunostained with rabbit anti-gp37 antibody (green). The expression levels of GADD45β (red) were analyzed using confocal microscopy. DAPI (blue) was used to stain the nuclear DNA. Scale bar, 10 μm. The chart (**c′**) shows the fluorescence intensity of endogenous GADD45β in (**c**). All experiments were repeated in triplicate.

Chickens with different genetic backgrounds demonstrate different susceptibilities to ALV-J; some breeds possess a stronger genetic resistance to ALV-J. In our previous studies^27^, the most resistant chickens were found to have significantly increased expression levels of the GADD45β gene in their livers, which suggested that the GADD45β gene may be closely related to ALV-J virus replication. To clarify the relationship between GADD45β and ALV-J, the expression levels of GADD45β in DF-1 cells following ALV-J infection were analyzed using numerous methods. Using RT-qPCR and western blotting, the expression levels of GADD45β were discovered to be increased in DF-1 cells at three different time points following ALV-J infection (*P* < 0.01, *P* < 0.001; Fig. 1a, b), what’s more, it’s also proved that ALV-J infection can increase the level of GADD45β protein in CEF cells. To further validate these findings, indirect immunofluorescence was used to detect the expression levels of endogenous GADD45β in DF-1 cells; the findings were consistent with the results obtained using RT-qPCR and western blotting (*P* < 0.05; Fig. 1c). These findings suggested that GADD45β expression levels may be increased following ALV-J infection.

### Both ALV-J infection and the overexpression of GADD45β inhibits autophagy

**Fig. 2:**
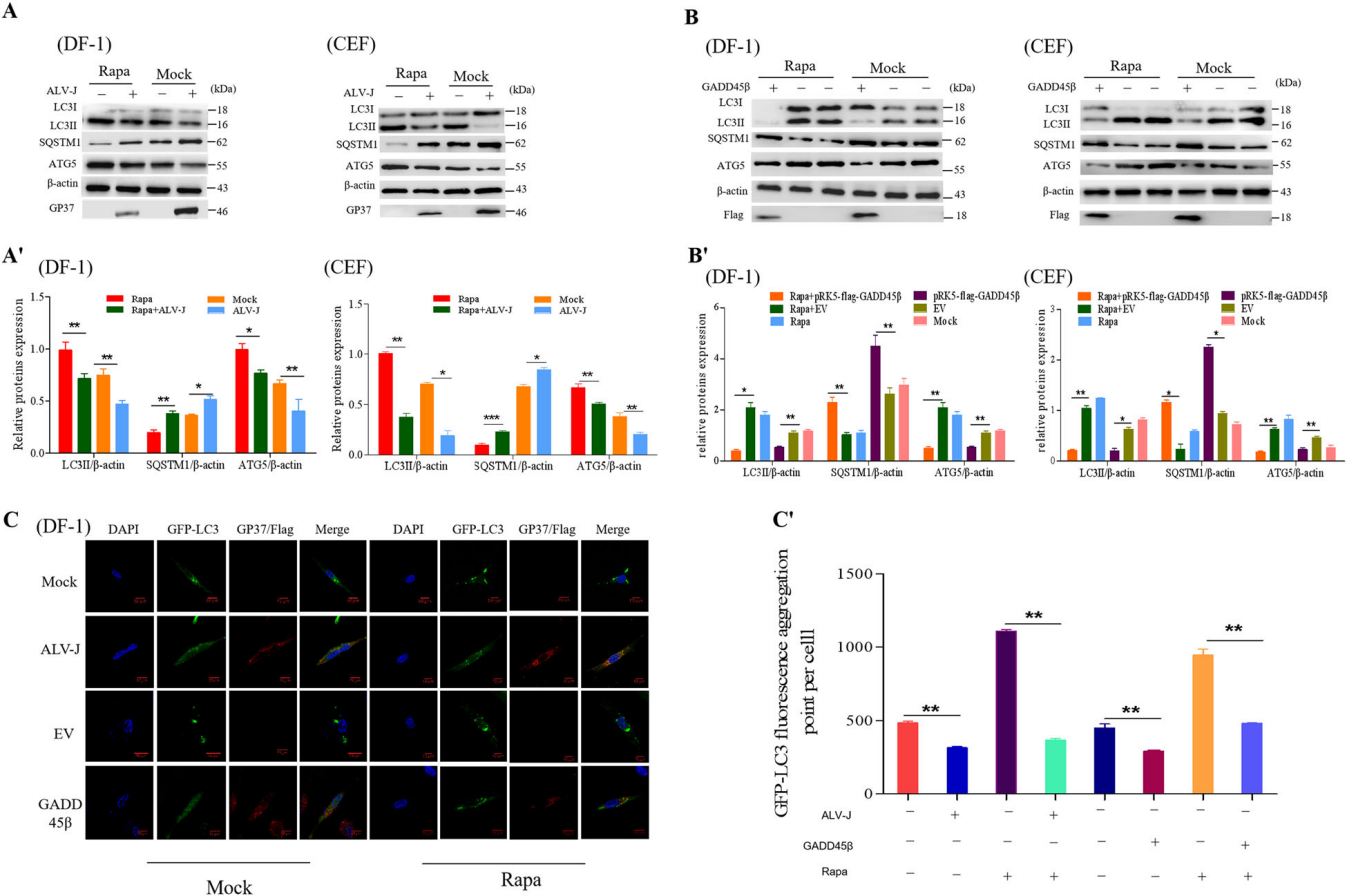
ALV-J infection and the overexpression of GADD45β inhibits autophagy. **a** DF-1 cells or CEF cells were pretreated with 1 μM Rapa for 2 h prior to being infected with ALV-J for 24 h. Expression levels of gp37, LC3B, SQSTM1, and Atg5 were analyzed using western blotting. The chart (**a′**) shows the quantification of LC3II/β-actin, SQSTM1/β-actin and Atg5/β-actin in (**a**). **b** DF-1 cells or CEF cells were transfected with a GADD45β overexpression plasmid and pretreated with Rapa. The expression levels of LC3, SQSTM1, and Atg5 were analyzed using western blotting. The chart (**b′**) shows the quantification of LC3II/β-actin, SQSTM1/β-actin and Atg5/β-actin in (**b**). **c** DF-1 cells were transfected with a GFP-LC3B plasmid for 18 h and either infected with ALV-J or transfected with pRK5-flag-GADD45β for 24 h separately. The number of GFP-LC3B-labeled autophagosomes (green) was observed using confocal microscopy in the presence or absence of Rapa stimulation. The chart (**c′**) shows the GFP-LC3 fluorescence aggregation point of per cell in (**c**). Data are presented as the mean ± SD of three independent experimental repeats.

It has been previously demonstrated that ALV-J inhibits autophagy and GADD45β is also well-known to negatively regulate autophagy. To investigate whether cellular macroautophagy was altered in response to ALV-J infection and the overexpression of GADD45β, the expression levels of autophagy-related proteins were analyzed in both DF-1 cells and CEF cells. The results indicated that the levels of endogenous lipidated LC3II in DF-1 cells, which correlates with an increased number of autophagosomes, were decreased compared with the mock-infected cells (*P* < 0.01; Fig. 2a, a′). Moreover, the expression levels of the autophagosome cargo SQSTM1, another autophagy marker, were markedly increased (*P* < 0.01). Expression levels of Atg5, an important factor involved in activating autophagosome formation and maturation^33^, were revealed to be decreased (*P* < 0.05). Based on the fact that Rapa induces autophagy, the appropriate concentration of Rapa was used to analyze the expression levels of LC3, SQSTM1, and Atg5 proteins; the expression levels of autophagy-related proteins followed the same trend observed following ALV-J infection. In addition, GFP-LC3 puncta can be a sign of enhanced autophagic flux or of impaired autophagosome lysosome fusion. Compared with mock-infected cells, the number of autophagosomes in the ALV-J infected cells transiently transfected with GFP-LC3B were decreased (*P* < 0.001; Fig. 2c, c′). Similarly, the recombinant plasmid pRK5-flag-GADD45β produced the same results (*P* < 0.001; Fig. 2b, c, c′). Thus, these findings suggested that both ALV-J infection and the overexpression of GADD45β may inhibit autophagy.

### Both ALV-J infection and the overexpression of GADD45β inhibits the autophagic flux

**Fig. 3:**
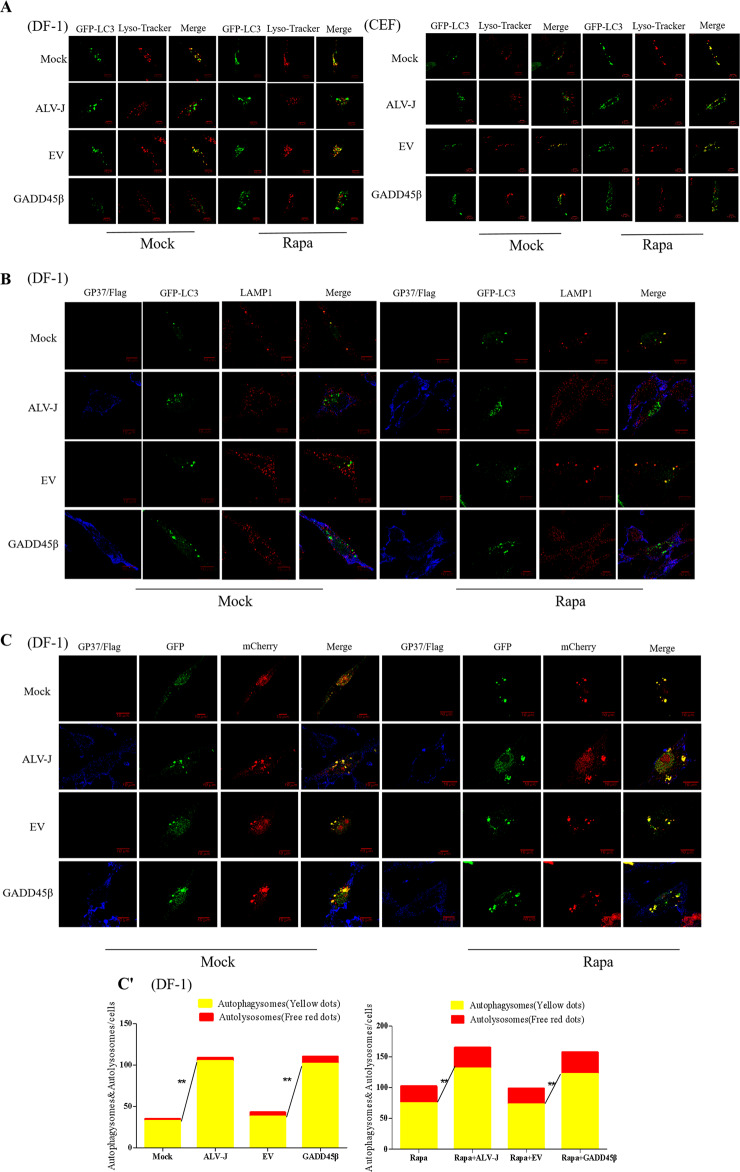
Autophagosomes are unable to fuse with lysosomes following ALV-J infection or the overexpression of GADD45β. **a** DF-1 cells or CEF cells were marked with Lyso Tracker Red (50 nM) for 2 h and GFP-LC3B-labeled autophagosomes (green) were visualized using confocal microscopy to colocalize red-stained acidified vesicles (red). **b** Cells were stained with anti-LAMP1 antibody (red) and anti-gp37 antibody (blue), and visualized using confocal microscopy to visualize the fusion between the autophagosomes and lysosomes. **c** Cells were infected with the mCherry-GFP-LC3B recombinant adenovirus to analyze the smoothness of autophagosome formation. Scale bar, 10 μm. The chart (**c′**) shows the quantification of GFP-mCherry-LC3 tandem reporter in (**c**).

Since autophagy is a dynamic process, the presence of GFP-LC3B-positive autophagosomes may suggest an increased rate of autophagy, either due to the accelerated formation of autophagosomes or by the accumulation of autophagosomes due to their reduced fusion with lysosomes^34^. To investigate the accumulation of autophagosomes during ALV-J infection, cells were labeled with LysoTracker Red to label acidic compartments or organelles in living cells. Autophagosomes and LysoTracker Red were not colocalized in DF-1 cells or CEF cells following ALV-J infection. Similarly, in ALV-J-infected cells treated with Rapa, almost no colocalization was observed between autophagosomes and LysoTracker Red. By contrast, autophagosomes colocalized with LysoTracker Red in the normal groups independent of the presence of absence of Rapa treatment (Fig. 3a). On the other hand, following the overexpression of GADD45β, autophagosomes were not colocalized with LysoTracker Red, which suggested that autophagosomes may not fuse with acidic compartments or organelles following the overexpression of GADD45β (Fig. 3a).

To rule out the probability that the autophagosomes fused with the lysosomes, but were not properly acidified in infected cells, the distributions of GFP-LC3B in relation to the protein, LAMP1, were investigated in ALV-J-infected cells and GADD45β overexpressing cells. Similarly, GFP-LC3B colocalized with LAMP1 in Rapa-treated and Rapa-untreated cells, and LAMP1 protein aggregated around the autophagosomes, whereas GFP-LC3B did not colocalize with LAMP1 in ALV-J-infected and GADD45β overexpressing cells (Fig. 3b). As the GFP green fluorescence will be quenched in the acidic environment of the lysosomes, which would increase the difficulty of tracking GFP-LC3B cell localization, mCherry-GFP-LC3B, expressing the fusion protein of red fluorescent protein mCherry, green fluorescent protein (GFP), and LC3B in target cells, was chosen to investigate the autophagy flux. Compared with the mock cells, following both ALV-J infection or GADD45β overexpression, the number of autophagosomes increased and the formation of yellow spots was observed (*P* < 0.01; Fig. 3c, c′). Altogether, these data indicated that ALV-J infection and the overexpression of GADD45β may inhibit the fusion of autophagosomes with lysosomes, thereby blocking the autophagic flux.

### GADD45β binding to MEKK4 is crucial for ALV-J infection by activating the p38MAPK signaling pathway

**Fig. 4:**
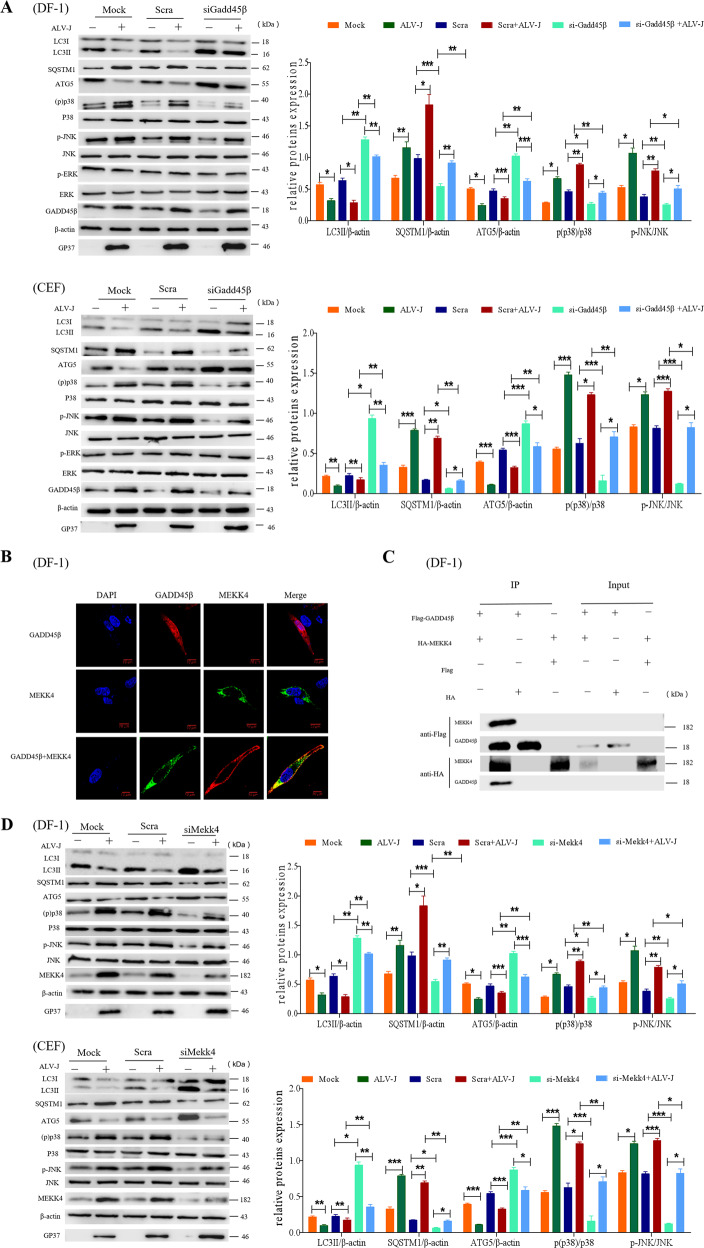
GADD45β interacts with MEKK4 in response to ALV-J infection-induced activation of the p38MAPK signaling pathway. **a** DF-1 cells or CEF cells were transfected with siRNA targeting GADD45β for 24 h and subsequently infected with ALV-J for 24 h. The infected cells were then collected for western blotting analysis of the expression levels of gp37, p-p38, p38, p-JNK, JNK, p-ERK, ERK, LC3B, SQSTM1, and Atg5. **b** DF-1 cells were co-transfected with Flag-GADD45β and HA-MEKK4 for 24 h and the colocalization of GADD45β (red) with MEKK4 (green) was detected using the corresponding antibodies under confocal microscopy. Scale bar, 10 μm. **c** DF-1 cells were co-transfected with Flag-GADD45β and HA-MEKK4 for 48 h, and the interaction between GADD45β and MEKK4 was determined using the indicated antibodies. **d** ALV-J infection was performed following the transfection with siRNA targeting MEKK4 in DF-1 cells or CEF cells for 24 h. Expression levels gp37, p-p38, p38, p-JNK, JNK, LC3B, SQSTM1, and Atg5 were analyzed using western blotting. Data are presented as the mean ± SD of three independent experimental repeats.

The GADD45β protein serves an important regulatory role in autophagy. It has been widely established that GADD45β actively regulates MAPK activity, including the p38MAPK, JNK, and ERK proteins. To investigate the role that GADD45β has in the incomplete autophagy pathway regulating ALV-J infection, the activation of p38MAPK, JNK, and ERK following ALV-J infection was investigated in DF-1 cells and CEF cells. Compared with the mock-infected cells, the expression levels of phosphorylated p38MAPK and JNK in ALV-J-infected cells were significantly increased (Fig. 4a, b), whereas the expression levels of p-ERK were not significantly different, suggesting that p38MAPK and JNK may be relevant to ALV-J infection. Hereafter, to investigate whether the p38MAPK and JNK signaling pathways were necessary steps in ALV-J-mediated autophagy, the p38MAPK and JNK signaling pathways were knocked down using siRNA targeting GADD45β prior to ALV-J infection. The results revealed that both p38MAPK and JNK phosphorylation levels were significantly decreased in cells treated with siRNA-GADD45β compared with the ALV-J-infected cells without siRNA interference, which suggested that GADD45β silencing may inhibit the phosphorylation of p38MAPK and JNK. To further determine the relationship between GADD45β, ALV-J and autophagy, the expression levels of LC3, SQSTM1, and Atg5 were investigated. In siRNA-GADD45β-transfected ALV-J-infected cells, the expression level of LC3II and Atg5 were significantly decreased, while SQSTM1 were significantly increased compared with ALV-J-infected cells without siRNA knockdown. Collectively, these observations indicated that GADD45β may be an upstream regulator of p38MAPK and JNK in ALV-J-mediated incomplete autophagy, and that ALV-J infection does not directly affect the phosphorylation status of p38MAPK and JNK.

MAP kinase kinase kinase, MTK1 (also known as MEKK4) has been identified as an interacting partner of GADD45β, which can activate not only p38MAPK, but also JNK. To further investigate the role of GADD45β and MEKK4 proteins in inhibiting autophagy, the interaction between GADD45β and MEKK4 in DF-1 cells was determined. Confocal analysis demonstrated that GADD45β strongly colocalized with MEKK4 in DF-1 cells co-transfected with the GADD45β and MEKK4 overexpression plasmids (Fig. 4c). In GADD45β and MEKK4-coexpressing cells, GADD45β demonstrated prominent cytosolic staining and colocalized with MEKK4, further validating that these two proteins are interaction partners. In addition, Co-IP analysis revealed that GADD45β potently interacted with MEKK4 in cells co-transfected with the GADD45β and MEKK4 overexpression plasmids (Fig. 4d). Moreover, the co-expression of GADD45β and MEKK4 resulted in significantly increased levels of phosphorylated p38 in DF-1 cells (Fig. S1). MEKK4 expression was genetically knocked down using siRNA to investigate whether ALV-J infection altered the expression levels of LC3, SQSTM1, and Atg5 in DF-1 cells or CEF cells (Fig. 4e). The results clearly demonstrated that LC3II and Atg5 expression levels were significantly decreased, whereas SQSTM1 expression levels were significantly increased in MEKK4-knockdown cells infected with ALV-J (*P* < 0.01). To confirm the upstream activity of MEKK4, it was investigated whether p38MAPK and JNK were activated. It was observed that the phosphorylation levels of p38MAPK and JNK were increased in normal cells compared with MEKK4-knockdown cells. Taken together, these data suggested that ALV-J may inhibit autophagy through the binding of GADD45β to MEKK4.

### GADD45β/MEKK4/p38MAPK signaling pathway is involved in autophagy inhibition during ALV-J infection

**Fig. 5:**
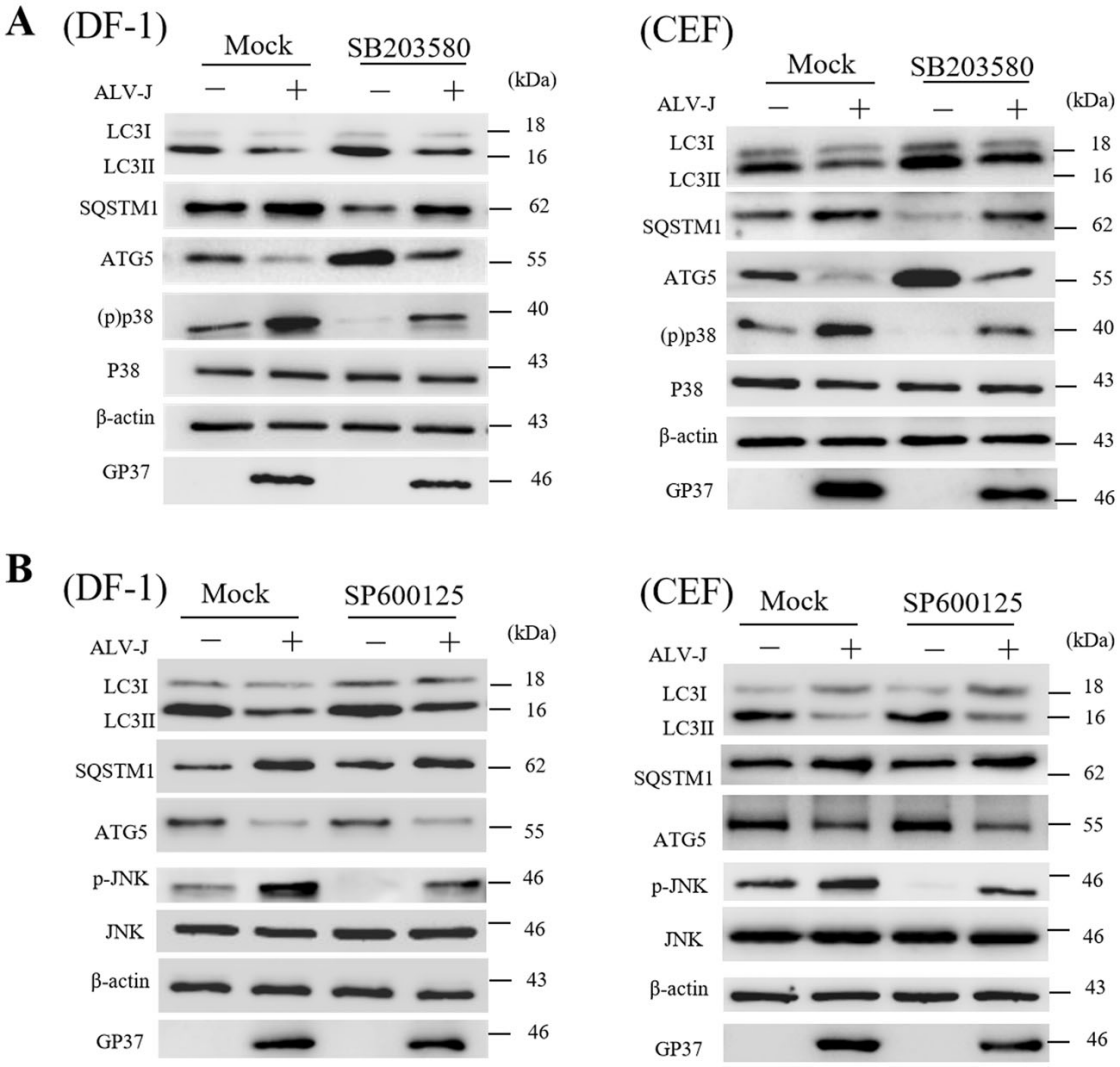
ALV-J inhibits autophagy through the GADD45β/MEKK4/p38MAPK signaling pathway. **a**, **b** DF-1 cells or CEF cells were treated with SB203580 and SP600125, inhibitors of the p38MAPK and JNK signaling pathways, respectively, and infected with ALV-J for 24 h. Expression levels of gp37, LC3B, SQSTM1, Atg5, p-p38, p38, p-JNK, and JNK were analyzed using western blotting.

SB203580 and SP600125, common p38MAPK and JNK inhibitors, respectively, markedly reduced the expression levels of p-p38 and p-JNK (Fig. S3A, B). Subsequently, the optional concentrations to inhibit the phosphorylation of p-p38 and p-JNK were chosen. The above experimental data indicated that ALV-J may inhibit autophagy through the interaction between GADD45β and MEKK4. Subsequently, the changes of autophagy protein expression levels were analyzed using western blotting following SB203580 and SP600125 treatment in DF-1-treated cells and CEF-treated cells. Compared with the mock-infected cells, the levels of phosphorylated p38MAPK were markedly decreased in SB203580-treated cells. The expression levels of LC3II and Atg5 were definitely increased, while SQSTM1 were significantly decreased in ALV-J-infected cells treated with SB203580 compared with ALV-J-infected cells (Fig. 5a), whereas there were no significant differences observed in SP600125-treated cells (Fig. 5b). Altogether, these findings suggested that ALV-J may inhibit autophagy through the GADD45β/MEKK4/p38MAPK signaling pathway, suggesting a novel role for p38MAPK in ALV-J infection autophagy regulation.

### Autophagy precedes apoptosis following ALV-J infection or the overexpression of GADD45β

**Fig. 6:**
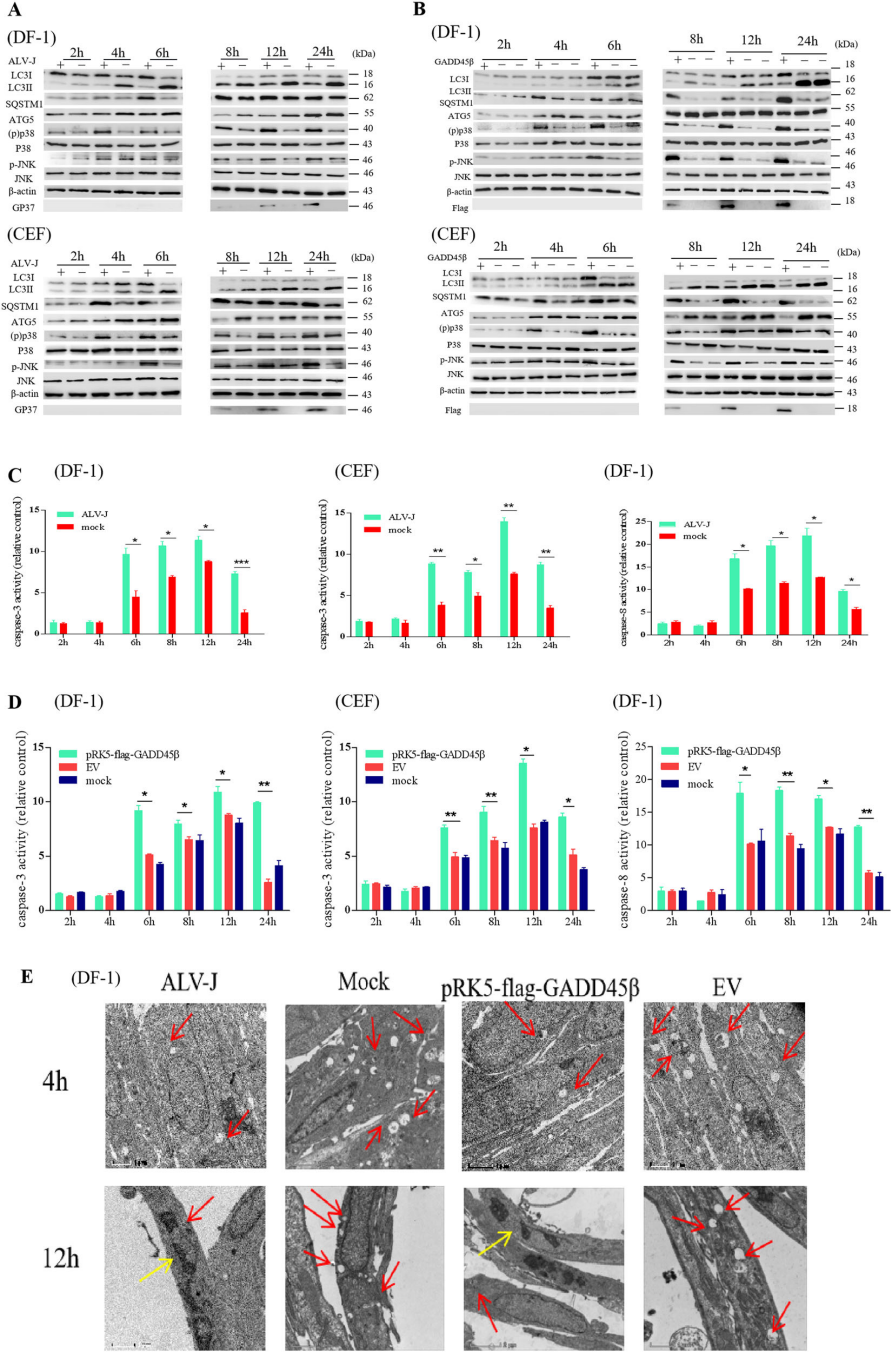
Autophagy precedes apoptosis following ALV-J infection or the overexpression of GADD45β. **a**, **b** Autophagy, but not apoptosis, was detected within 4 h of ALV-J infection or GADD45β overexpression. The expression levels of relevant important autophagy proteins and signaling pathway proteins (gp37, LC3B, SQSTM1, Atg5, p38, JNK, p38, and p-JNK) were detected using western blotting following ALV-J infection or GADD45β overexpression for 2, 4, 6, 8, 12, and 24 h. **c**, **d** Caspase-3 and caspase-8 activity assay kits were used to analyze caspase-3 and caspase-8 enzyme activities in DF-1 cells or CEF cells following ALV-J infection or GADD45β overexpression for 2, 4, 6, 8, 12, and 24 h. **e** As discovered through TEM analysis, autophagic compartments (autophagosomes and autolysosomes), but not apoptotic features, were present 4 h after ALV-J infection or GADD45β overexpression. However, after ALV-J infection or GADD45β overexpression for 12 h, not only were autophagic compartments present, but apoptotic features were also observed in some DF-1 cells. Red arrow indicates an autophagosome or autolysosome. Yellow arrows denote the abnormal morphology of the nuclear membrane.

Due to both p38MAPK and JNK serving pivotal roles in the crosstalk between autophagy and apoptosis, and the above experimental data indicating that both the p38MAPK and JNK signal pathways are activated following ALV-J infection, it was investigated whether autophagy may precede apoptosis. Interestingly, the expression levels of autophagy-related proteins of LC3II (*P* < 0.01) and Atg5 were definitely increased (*P* < 0.001), while SQSTM1 were significantly decreased (*P* < 0.05) following the infection with ALV-J or the overexpression of GADD45β for 4 h in DF-1 cells or CEF cells. The activation of p38MAPK was also detected. Notably, the inhibition of autophagy and the activation of p38MAPK were observed within 4 h of ALV-J infection or the overexpression of GADD45β, and persisted for 24 h in both DF-1 cells and CEF cells (Fig. 6a, b). In addition, following 6 h of ALV-J infection or the overexpression with GADD45β, caspase-3 activities were observed to be activated in both DF-1 cells and CEF cells (Fig. 6c), and caspase-8 activities were also to be activated at the same time (Fig. 6d). Unexpectedly, JNK was also activated within 6 h of ALV-J infection or the overexpression of GADD45β (*P* < 0.05; Fig. 6a, b), and it was continuously activated for 24 h. Therefore, it was demonstrated that the inhibition of autophagy preceded the induction of apoptosis in DF-1 cells following ALV-J infection or the overexpression of GADD45β. Thus, ALV-J infection or the overexpression of GADD45β may activate the p38MAPK and JNK signaling pathways and p38MAPK is activated prior to JNK.

Finally, whether ALV-J infection or the overexpression of GADD45β affected autophagy and apoptosis in the same DF-1 cells was investigated. TEM analysis revealed the presence of autophagic compartments, but without apoptotic features, at 4 h following ALV-J infection and the overexpression of GADD45β, and the number of autophagosomes formed were decreased compared with the normal group. Nevertheless, both autophagic compartments and apoptotic features were observed in a number of DF-1 cells at 12 h post ALV-J infection or following the overexpression of GADD45β, with the nucleus ruptured into several segments and no intact nuclear morphology (Fig. 6e). Our previous experimental data demonstrated that both ALV-J infection or the overexpression of GADD45β inhibited autophagy faster than induced apoptosis in DF-1 cells and CEF cells.

## Discussion

Research into the regulatory roles of autophagy has become an increasingly popular topic in numerous research fields, including its regulation over cancer drug resistance and development^35^, degenerative diseases^36^, lipid metabolism^37^, and the control of viral pathogenesis and immunology^38^. Autophagy can directly eliminate intracellular pathogens, including bacteria and viruses, by encapsulation, phagocytosis and lysosomal degradation. However, viruses can not only interact with immune receptors, membrane surface receptors and autophagy-related proteins, but they also affect the environmental stress of intracellular cells through affecting autophagic processes to promote virus survival or infection^39^. The exploitation of autophagy has been identified in several RNA viruses, including PV, CVB3, JEV, and HCV^40–43^. In the present study, a reduction in the number of autophagosomes formed and an evident increase in SQSTM1 expression levels were observed in ALV-J-infected cells. In addition, cells treated with an autophagy stimulant as a control were able to induce autophagy, further suggesting that ALV-J may block the early stages of the autophagic pathway. Also, GFP-LC3B was not colocalized with LysoTracker Red or LAMP1 (a lysosomal marker) in ALV-J-infected cells (Fig. 3). These findings indicated that ALV-J infection may inhibit the early stages of autophagy, preventing the autophagosomes from fully fusing with the lysosomes, and resulting in an impeded autophagy flux.

GADD45β is an ordinary regulatory protein that controls autophagy and apoptosis. The interference of GADD45β expression combined with the treatment with p38 inhibitors has been reported to significantly induce autophagy^44^. In addition, GADD45β has been found to be induced upon partial hepatectomy, which inhibited pro-apoptotic JNK signaling^45^. In the present study, the overexpression of GADD45β in DF-1 cells inhibited autophagy and promoted apoptosis, in addition to increasing the expression levels of JNK following the activation of p38MAPK at different time intervals. GADD45β performs its physiological functions through protein–protein interactions in the nucleus and cytoplasm, modulating cell proliferation, cell death, and cell survival^46^. Interestingly, GADD45β expression was located in both the nucleus and cytosol when expressed alone, whereas upon the co-expression of GADD45β and MEKK4, GADD45β demonstrated prominent cytoplasmic staining, where it was found colocalized with MEKK4. This also increased p38MAPK expression levels. In an inactivated state, MEKK4 normally exists in a closed conformation and only undergoes dimerization, autophosphorylation and subsequent activation of downstream kinases after binding to the GADD5β protein^47^. In a previous study, GADD45β and MEKK4-activated p38MAPK was observed to prevent the fusion of autophagosomes with lysosomes^26^, which is consistent with the findings of the present study, whereby ALV-J infection inhibited the fusion of the autophagosomes with the lysosomes. A previous study has also shown that hepatitis C virus promoted cell cycle arrest by decreasing GADD45β expression levels, indicating that GADD45β is tightly related by the hepatitis C virus^48^. Our previous study also demonstrated that GADD45β affected ALV-J virus replication^49^. In the current study, an association between ALV-J infection and increased expression levels of GADD45β was revealed. The data revealed that ALV-J inhibited autophagy through activating the GADD45β/MEKK4/p38MAPK signaling pathway following GADD45β binding to MEKK4.

Recent studies have reported that MAPKs, in particular p38MAPK and JNK, serve an important role in the crosstalk between autophagy and apoptosis. Regarding p38MAPK, both autophagy promoting functions and suppressing functions have been reported; the accumulation of glial fibrillary acidic protein activated p38ΜΑPK, which directly inhibited mTOR and induced autophagy^50^. GABARAP, a mammalian Atg8 homolog, is upregulated following the pharmacological inhibition of p38ΜΑPK in a colon cancer cell line, resulting in autophagy and cell death^51^. In addition, MAPK8/JNK1, which phosphorylates Bcl-2 releasing Beclin-1^52^, has been observed to promote autophagy as part of the class III PtdIns3K complex^53^. In the present study, both p38MAPK and JNK were simultaneously activated. The accumulation of SQSTM1 protein indicated that ALV-J may block the early stages of the autophagic pathway, and it was further demonstrated that ALV-J infection inhibited autophagy through activating the GADD45β/MEKK4/p38ΜΑPK signaling pathway instead of the GADD45β/MEKK4/JNK signaling pathway. Previous studies have reported that JNK initiated the phosphorylation of c-Jun and ATF-2, and mediated caspase-8 and caspase-3 activation to trigger apoptosis^54^. Similarly, the current study detected increases in caspase-8 and caspase-3 activities following ALV-J infection or the overexpression of GADD45β. Thus, it was hypothesized that ALV-J may activate caspase-8 and caspase-3 to trigger apoptosis through the GADD45β/MEKK4/JNK signaling pathway. Nevertheless, the exact mechanism remains poorly understood and requires further studies in the future.

The interaction between apoptosis and autophagy may be more perplexing than previously thought^55^, as it has been suggested that they may share common molecular inducers and regulatory mechanisms^56–58^, which may coexist or occur sequentially. For example, during the metamorphosis of silkworms, autophagy and apoptosis presented simultaneously in the fat body^59^ and silk gland^60^, whereas autophagy preceded apoptosis in the midgut cells^61^. In mammals, SQSTM1 and Atg5 have been discovered to be molecular switches that trigger apoptosis during autophagy in response to stress conditions. SQSTM1 interplayed with polyubiquitinated caspase-8, which subsequently promoted the oligomerization and activation of caspase-8^62^. In addition, the Atg12-Atg5 complex interacted with FADD to recruit caspase-8 and induce apoptosis. Therefore, the expression levels of SQSTM1 and Atg5, and the activity of caspase-8 were analyzed at six different time points during ALV-J infection and it was discovered that the expression levels of SQSTM1 and Atg5 changed before caspase-8 activity. TEM analysis also revealed that ALV-J infection inhibited autophagy prior to the induction of apoptosis. It is evident that autophagy can promote cell death; both p38MAPK and JNK were successfully activated during ALV-J infection, of which both can affect apoptosis and autophagy in response to extracellular stimulation. Nevertheless, it remains unknown how cells react to similar stimuli by preferentially undergoing apoptosis or autophagy. The present study findings may offer novel insights into the role of ALV-J infection in controlling the balance between autophagy and apoptosis.

It was discovered that ALV-J infection activates the GADD45β/MEKK4/p38MAPK signaling pathway to inhibit autophagy through the interaction between GADD45β and MEKK4. In fact, however, the expression levels of GADD45β, or the rate of autophagy and apoptosis, changed in response to ALV-J infection, research should focus on the ALV-J virus itself. Thus, further research is required to determine which component (viral protein, nucleotides or other substances) of ALV-J affects GADD45β expression, in addition to the activation of p38MAPK and JNK.

In conclusion, the present study investigated the effects of ALV-J infection on autophagy and apoptosis, and determined a potential mechanism by which ALV-J infection may regulate autophagy through the GADD45β/MEKK4/p38MAPK signaling pathway.

## Supplementary information

Figure S1

Figure S2

Figure S3

## References

[1] Gabryel B, Kost A, Kasprowska D (2012). Neuronal autophagy in cerebral ischemia-a potential target for neuroprotective strategies?. Pharmacol. Rep..

[2] Erlich S (2007). Differential interactions between Beclin 1 and Bcl-2 family members. Autophagy.

[3] Bell BD (2008). FADD and caspase-8 control the outcome of autophagic signaling in proliferating T cells. Proc. Natl Acad. Sci. USA.

[4] Pyo JO (2005). Essential roles of Atg5 and FADD in autophagic cell death: dissection of autophagic cell death into vacuole formation and cell death. J. Biol. Chem..

[5] Radoshevich L (2010). ATG12 conjugation to ATG3 regulates mitochondrial homeostasis and cell death. Cell.

[6] Booth LA (2014). The role of cell signalling in the crosstalk between autophagy and apoptosis. Cell Signal..

[7] Sun D (2019). Apoptosis and Autophagy in Picornavirus Infection. Front. Microbiol..

[8] Pei J (2014). Autophagy enhances the replication of classical swine fever virus in vitro. Autophagy.

[9] Sun MX (2012). Porcine reproductive and respiratory syndrome virus induces autophagy to promote virus replication. Autophagy.

[10] Pei J (2014). Autophagy enhances the replication of classical swine fever virus in vitro. Autophagy.

[11] Guo X (2017). Porcine epidemic diarrhea virus induces autophagy to benefit its replication. Viruses.

[12] Ma Y, Galluzzi L, Zitvogel L, Kroemer G (2013). Autophagy and cellular immune responses. Immunity.

[13] Kyei GB (2019). Autophagy pathway intersects with HIV-1 biosynthesis and regulates viral yields in macrophages. J. Cell Biol..

[14] Fields J (2013). Alterations in the levels of vesicular trafficking proteins involved in HIV replication in the brains and CSF of patients with HIV-associated neurocognitive disorders. J. Neuroimmune Pharmacol..

[15] Chauhan S, Mandell MA, Deretic V (2015). IRGM governs the core autophagy machinery to conduct antimicrobial defense. Mol. Cell.

[16] Liu H (2013). Subgroup J avian leukosis virus infection inhibits autophagy in DF-1 cells. Virol. J..

[17] Csibi A, Blenis J (2012). Hippo-YAP and mTOR pathways collaborate to regulate organ size. Nat. Cell Biol..

[18] Li H (2014). gga-miR-375 plays a key role in tumorigenesis post subgroup J avian leukosis virus infection. PLoS ONE.

[19] Liang N (2014). Regulation of YAP by mTOR and autophagy reveals a therapeutic target of tuberous sclerosis complex. J. Exp. Med..

[20] Feng SZ, Cao WS, Liao M (2011). The PI3K/Akt pathway is involved in early infection of some exogenous avian leukosis viruses. J. Gen. Virol..

[21] Liebermann DA, Hoffman B (2003). Myeloid differentiation (MyD) primary response genes in hematopoiesis. Blood Cells Mol. Dis..

[22] Koonin EV (1997). Cell cycle and apoptosis: possible roles of Gadd45 and MyD118 proteins inferred from their homology to ribosomal proteins. J. Mol. Med..

[23] Gupta M (2005). Hematopoietic cells from Gadd45a- and Gadd45b-deficient mice are sensitized to genotoxic-stress-induced apoptosis. Oncogene.

[24] Lu B, Ferrandino AF, Flavell RA (2004). Gadd45beta is important for perpetuating cognate and inflammatory signals in T cells. Nat. Immunol..

[25] Papa S (2004). Gadd45 beta mediates the NF-kappa B suppression of JNK signalling by targeting MKK7/JNKK2. Nat. Cell Biol..

[26] Keil E (2013). Phosphorylation of Atg5 by the Gadd45beta-MEKK4-p38 pathway inhibits autophagy. Cell Death Differ..

[27] Zhang X (2016). GADD45beta, an anti-tumor gene, inhibits avian leukosis virus subgroup J replication in chickens. Oncotarget.

[28] Han J, Lee JD, Bibbs L, Ulevitch RJA (1994). MAP kinase targeted by endotoxin and hyperosmolarity in mammalian cells. Science.

[29] Cuenda A, Rousseau S (2007). p38 MAP-kinases pathway regulation, function and role in human diseases. Biochim Biophys. Acta.

[30] de la Cruz-Morcillo MA (2012). P38MAPK is a major determinant of the balance between apoptosis and autophagy triggered by 5-fluorouracil: implication in resistance. Oncogene.

[31] Cruz-Morcillo MA, Sanchez-Prieto R (2012). Autop38-phagy and apop38-tosis in genotoxic stress: a strange duo. Autophagy.

[32] Webber JL, Tooze SA (2010). Coordinated regulation of autophagy by p38alpha MAPK through mAtg9 and p38IP. Embo J..

[33] Matsushita M (2007). Structure of Atg5.Atg16, a complex essential for autophagy. J. Biol. Chem..

[34] Galluzzi L (2018). Molecular mechanisms of cell death: recommendations of the Nomenclature Committee on Cell Death 2018. Cell Death Differ..

[35] Mahoney E, Byrd JC, Johnson AJ (2013). Autophagy and ER stress play an essential role in the mechanism of action and drug resistance of the cyclin-dependent kinase inhibitor flavopiridol. Autophagy.

[36] Yang Y (2014). Stimulation of autophagy prevents amyloid-beta peptide-induced neuritic degeneration in PC12 cells. J. Alzheimers Dis..

[37] Saito T (2019). Autophagy regulates lipid metabolism through selective turnover of NCoR1. Nat. Commun..

[38] Jackson WT (2005). Subversion of cellular autophagosomal machinery by RNA viruses. PLoS Biol..

[39] Grose C, Klionsky DJ (2016). Alternative autophagy, brefeldin A and viral trafficking pathways. Autophagy.

[40] Jackson WT (2005). Subversion of cellular autophagosomal machinery by RNA viruses. PLoS Biol..

[41] Wong J (2008). Autophagosome supports coxsackievirus B3 replication in host cells. J. Virol..

[42] Tanida I (2009). Knockdown of autophagy-related gene decreases the production of infectious hepatitis C virus particles. Autophagy.

[43] Li JK, Liang JJ, Liao CL, Lin YL (2012). Autophagy is involved in the early step of Japanese encephalitis virus infection. Microbes Infect..

[44] He G (2016). Gadd45b prevents autophagy and apoptosis against rat cerebral neuron oxygen-glucose deprivation/reperfusion injury. Apoptosis.

[45] Papa S (2008). Gadd45beta promotes hepatocyte survival during liver regeneration in mice by modulating JNK signaling. J. Clin. Investig..

[46] Gerwins P, Blank JL, Johnson GL (1997). Cloning of a novel mitogen-activated protein kinase kinase kinase, MEKK4, that selectively regulates the c-Jun amino terminal kinase pathway. J. Biol. Chem..

[47] Miyake Z, Takekawa M, Ge Q, Saito H (2007). Activation of MTK1/MEKK4 by GADD45 through induced N-C dissociation and dimerization-mediated trans autophosphorylation of the MTK1 kinase domain. Mol. Cell Biol..

[48] Higgs MR, Lerat H, Pawlotsky JM (2010). Downregulation of Gadd45beta expression by hepatitis C virus leads to defective cell cycle arrest. Cancer Res..

[49] Zhang X (2016). GADD45beta, an anti-tumor gene, inhibits avian leukosis virus subgroup J replication in chickens. Oncotarget.

[50] Cheon SY, Cho KJ, Song J, Kim GW (2016). Knockdown of apoptosis signal-regulating kinase 1 affects ischaemia-induced astrocyte activation and glial scar formation. Eur. J. Neurosci..

[51] Comes F (2007). A novel cell type-specific role of p38alpha in the control of autophagy and cell death in colorectal cancer cells. Cell Death Differ..

[52] Barutcu SA, Girnius N, Vernia S, Davis RJ (2018). Role of the MAPK/cJun NH2-terminal kinase signaling pathway in starvation-induced autophagy. Autophagy.

[53] Ma X (2017). MTORC1-mediated NRBF2 phosphorylation functions as a switch for the class III PtdIns3K and autophagy. Autophagy.

[54] Balduini W, Carloni S, Buonocore G (2009). Autophagy in hypoxia-ischemia induced brain injury: evidence and speculations. Autophagy.

[55] Balduini W, Carloni S, Buonocore G (2012). Autophagy in hypoxia-ischemia induced brain injury. J. Matern Fetal Neonatal Med..

[56] Koike M (2008). Inhibition of autophagy prevents hippocampal pyramidal neuron death after hypoxic-ischemic injury. Am. J. Pathol..

[57] Lalaoui N, Lindqvist LM, Sandow JJ, Ekert PG (2015). The molecular relationships between apoptosis, autophagy and necroptosis. Semin Cell Dev. Biol..

[58] Sekimoto T, Iwami M, Sakurai S (2006). Coordinate responses of transcription factors to ecdysone during programmed cell death in the anterior silk gland of the silkworm, Bombyx mori. Insect Mol. Biol..

[59] Tian L (2013). 20-Hydroxyecdysone upregulates Atg genes to induce autophagy in the Bombyx fat body. Autophagy.

[60] Franzetti E (2012). Autophagy precedes apoptosis during the remodeling of silkworm larval midgut. Apoptosis.

[61] Pan JA, Ullman E, Dou Z, Zong WX (2011). Inhibition of protein degradation induces apoptosis through a microtubule-associated protein 1 light chain 3-mediated activation of caspase-8 at intracellular membranes. Mol. Cell Biol..

[62] Jin Z (2009). Cullin3-based polyubiquitination and p62-dependent aggregation of caspase-8 mediate extrinsic apoptosis signaling. Cell.

