# Supplementary figures and images for: ALV-J inhibits autophagy through the GADD45β/MEKK4/P38MAPK signaling pathway and mediates apoptosis following autophagy
Source: Cell Death Dis. 2020 Aug 12;11(8):684. doi: 10.1038/s41419-020-02841-y (PMC7442830; doi:10.1038/s41419-020-02841-y)

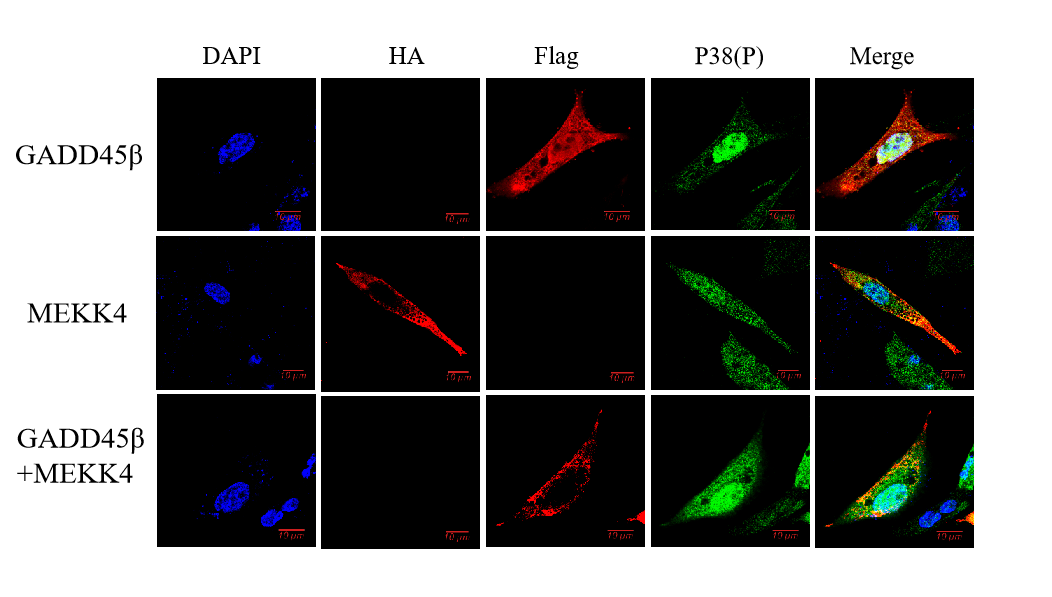

Supplement: Supplementary file 1 — Figure S1 [file 41419_2020_2841_MOESM1_ESM.tif]

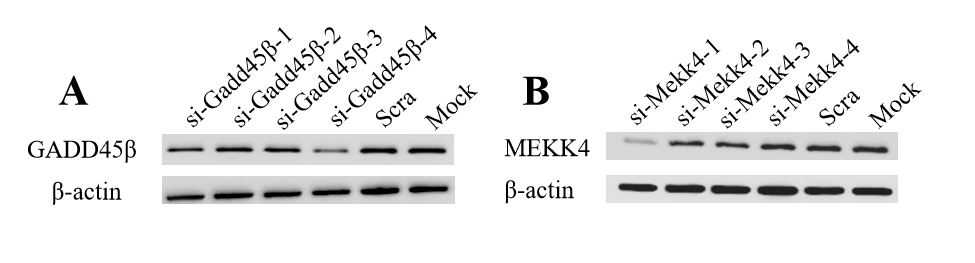

Supplement: Supplementary file 2 — Figure S2 [file 41419_2020_2841_MOESM2_ESM.tif]

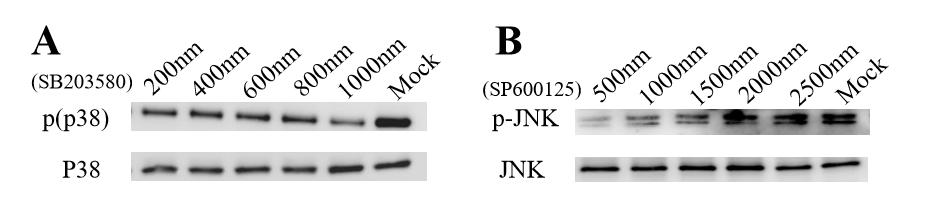

Supplement: Supplementary file 3 — Figure S3 [file 41419_2020_2841_MOESM3_ESM.tif]
